# Fresnel Diffraction Model for Laser Dazzling Spots of Complementary Metal Oxide Semiconductor Cameras

**DOI:** 10.3390/s24175781

**Published:** 2024-09-05

**Authors:** Xinyu Wang, Zhongjie Xu, Hairong Zhong, Xiang’ai Cheng, Zhongyang Xing, Jiangbin Zhang

**Affiliations:** 1 College of Advanced Interdisciplinary Studies, National University of Defense Technology, Changsha 410073, China; wangxinyu18@nudt.edu.cn (X.W.); xuzhongjie11@nudt.edu.cn (Z.X.); 13975148798@163.com (H.Z.); xiang_ai_cheng@126.com (X.C.); 2 State Key Laboratory of Pulsed Power Laser Technology, Changsha 410073, China; 3 Hunan Provincial Key Laboratory of High Energy Laser Technology, Changsha 410073, China

**Keywords:** Fresnel diffraction, FSI-CMOS, BSI-CMOS, laser dazzling

## Abstract

Laser dazzling on complementary metal oxide semiconductor (CMOS) image sensors is an effective method in optoelectronic countermeasures. However, previous research mainly focused on the laser dazzling under far fields, with limited studies on situations that the far-field conditions were not satisfied. In this paper, we established a Fresnel diffraction model of laser dazzling on a CMOS by combining experiments and simulations. We calculated that the laser power density and the area of saturated pixels on the detector exhibit a linear relationship with a slope of 0.64 in a log-log plot. In the experiment, we found that the back side illumination (BSI-CMOS) matched the simulations, with an error margin of 3%, while the front side illumination (FSI-CMOS) slightly mismatched the simulations, with an error margin of 14%. We also found that the full-screen saturation threshold for the BSI-CMOS was 25% higher than the FSI-CMOS. Our work demonstrates the applicability of the Fresnel diffraction model for BSI-CMOS, which provides a valuable reference for studying laser dazzling.

## 1. Introduction

Complementary metal oxide semiconductor (CMOS) image sensors are widely applied in various fields, including communications [1], autonomous driving [2], medical imaging [3], industrial automation [4], astronomy [5,6], and military applications [7]. As the core component of an electro-optical imaging system, the image sensor strongly absorbs laser energy within its operating wavelength range, making it more susceptible to laser damage compared to other parts of the electro-optical imaging system [8]. When the light intensity is high, the sensor is influenced in two ways, namely soft damage and hard damage [9]. Soft damage, or dazzle, usually refers to transient and reversible changes in device function, such as a decrease in device sensitivity, an increase in crosstalk, or a temporary failure of the device due to changes in material properties. Common laser dazzling phenomena in CMOS sensors include saturation, oversaturation [10,11,12], cross-talk [13,14,15], and full-screen saturation [16,17]. Although damaging sensors with lasers may be an alternative method, it is often difficult due to the need of high-power short-pulse lasers [7]. However, the power density required for dazzling is several orders of magnitude lower compared to image sensor damage [18]. Thus, fully damaging the image sensor is rather difficult [19,20]. Here, we focus on the irradiation effect and, especially, the dazzle effect.

When irradiated with lasers, the pixels in a CMOS image sensor can become saturated. Due to diffraction and scattering effects, as the power density of the incident laser increase, the saturation area of the sensor expands. Therefore, the degree of dazzle on CMOS can be described the laser dazzling area or the number of saturated pixels. In 2007, Schleijpen et al. found that under low mid-infrared laser irradiation, the radius of saturated spots and the laser irradiation exhibited a slope of 1/3 in the double logarithmic coordinates. At higher irradiation levels, the slope approached 1/2; the reason for this phenomenon had not been explained [21]. In 2010, Jiang et al. studied such relationship in visible charge coupled device (CCD) cameras, which gave a slope of 0.67 with validation of Fraunhofer diffraction model [22]. In the same year, Han et al. demonstrated this slope under intense illumination [23]. The quantitative relationship between the saturated area *S* and the incident light power *P* is described by Equation (1) [23]. In 2018, Yang et al. found that the diffraction effect of the surface optical system is the main cause of the expansion of the detector’s saturated area, closely related to parameters such as incident light intensity, wavelength, optical system parameters, and saturation threshold [24].

(1) $$\mathrm{lg}S=\mathrm{lg}\left[\frac{1}{\pi }{\left(\frac{\lambda z}{RI_{th}}\right)}^{\frac{2}{3}}\right]+\frac{2}{3}\mathrm{lg}P$$

When a high-density laser beam with wavelength λ and a radius of the diffraction pupil *R* is incident on a detector, located at a distance *z* from the diffraction pupil, the saturation threshold of the detector is denoted by $I_{\mathrm{th}}$, and the incident laser power on the detector is *P*.

In addition to diffraction, the impact of scattering on sensors under high-irradiation is of great importance. In 2011, Sun et al. studied the distribution of stray light under strong light irradiation [25]. In 2014, Wang et al. investigated the dazzle and damage effects of continuous lasers on CMOS, proposing a method to evaluate laser dazzle effects [26]. Koen et al. described a model for predicting the area of over-exposed pixels in CCD cameras under continuous wave (CW) illumination considering optical effects [27]. This model is accurate at low power density, but at higher power density, scattering led to a mismatch between experimental results and model predictions. In 2017, Niu et al. derived a theoretical model for laser dazzle flare, and showed that both the main and secondary flare spots appeared as regular circular patterns as the light intensity increased. Good agreement between model and experiment was achieved [28]. In 2022, Gareth et al. discovered that the diameter of saturated spots and laser power density exhibited different quantitative relationships under various integration times, where results of an integration time of 0.741 ms were consistent with Airy disc diffraction [29].

When the laser is acting in the far field, there are many factors that affect the number of saturated pixels on the sensor. In general, CCD cameras are more sensitive to dazzling when compared with CMOS cameras. In 2019, Santos et al. used visible and near-infrared lasers to dazzle CCD and CMOS cameras, including wide field of view (WFOV) optics, commonly found in smaller UAVs. The influence of various parameters was studied, such as laser wavelength, laser power, and angle of incidence [30]. Özbilgin et al. provided a unified framework for evaluating the number of glare pixels on camera sensors, taking into account a range of parameters including lasers, beam directors, sensors, optical cameras, and atmospheric parameters [31]. However, under laboratory conditions, the far-field criteria are not always fully met. When the distance between the laser and the detector is too short, it becomes difficult to determine whether the expanded laser beam satisfies the far-field conditions. Consequently, existing theories may not adequately explain certain experimental results, necessitating the development of a new diffraction model to account for these phenomena.

In this paper, we conducted the experiments using a 532 nm continuous laser to dazzle two types of CMOS image sensors, including a front side illumination CMOS (FSI-CMOS) and a backside illumination CMOS (BSI-CMOS). We captured images of laser dazzling for both CMOS image sensors at different power density and calculated the full-screen saturation threshold. Then, we obtained the quantitative relationship between the saturated spots area and entrance pupil power density. Finally, we built a simulation model based on the Fresnel diffraction and demonstrated the applicability of the model with experimental results.

## 2. Experimental Set-Up

**Figure 1:**
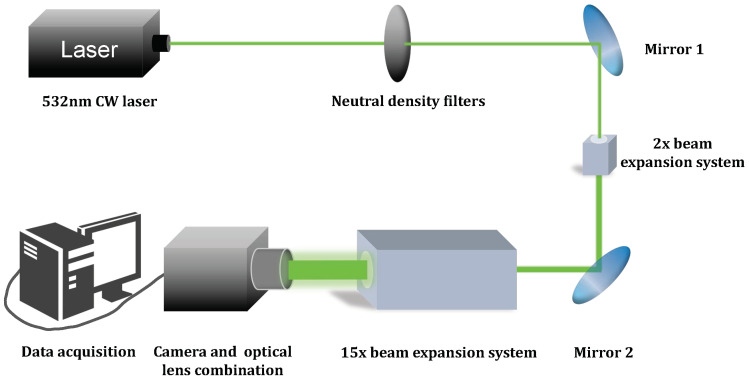
Schematic diagram of the optical path of CMOS image sensors dazzled by a 532 nm laser.

In this experiment, we used a 532 nm CW laser to radiate a FSI-CMOS camera and a BSI-CMOS camera with a transmissive commercial lens. The schematic diagram of the experimental optical path is shown in Figure 1.

The laser used in this study is a highly stable 532 nm solid-state CW laser with a maximum power output of 3 W. It can emit in the TEM_00_ mode with a beam diameter of approximately 3 mm. The specific parameters of the laser are shown in Table 1.

As shown in Figure 1, the laser had an output power of 500 mW incident into a series of neutral density (ND) filters, which can tune the laser power density for the following optical path. The working wavelength of the ND filters ranged from 400 nm to 650 nm. To reduce the influence of ghost images [32] on the dazzle experiment, we maintained a distance of approximately 3 cm between filters. The attenuated laser was then reflected by mirror 1 (silver-coated, reflection > 96%), and went through a 2× beam expansion system, outputting a laser beam with the diameter expanded to 6 mm. Finally, the laser radiated into the camera through the optical lenses.

**Figure 2:**
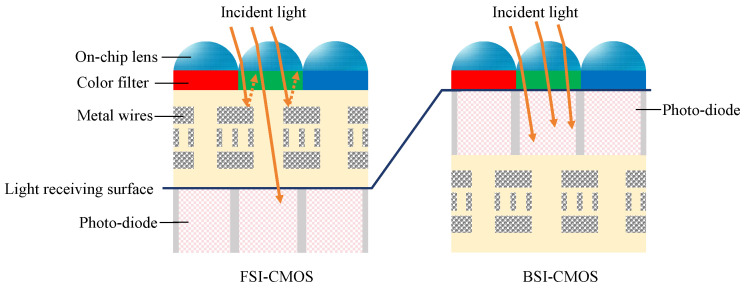
Pixel structures comparison of FSI-CMOS and BSI-CMOS [8].

The structures of FSI-CMOS camera and BSI-CMOS camera types are shown in Figure 2 [8].

The primary difference between front- and back-illumination types is the location of the photo-diode layer, which is in the front of the circuit layer for the former one while behind for the latter. In traditional FSI-CMOS sensors, light first passes through the circuit layer in order to reach the photo-diode layer. Therefore, some light may be absorbed or reflected by the circuit layer, ending up with reduction in the image brightness and clarity. In contrast, BSI-CMOS sensors, due to their unique design, show higher photo-sensitivity and image quality under weak-light conditions.

The two types of image sensors we used have similar parameters, as shown in Table 2. The quantum efficiency is over 70% at 532 nm for both sensors. The maximum laser power that can radiate into the camera is approximately 22 mW, which is well below the damage threshold of the camera’s focal plane. The maximum allowable gray value for images captured by this camera is 65,535.

Additionally, we placed a set of transmission lenses to focus the laser spots on the detector. The focal length of the lens is 35 mm and the aperture size is 25 mm. During the experiment, integration time of the camera was fixed to 6 ms for comparison. The laser power density at the entrance pupil ranged from $1\times 10^{-12}$ W/cm^2^ to $3\times 10^{-3}$ W/cm^2^. The camera did not show any sign of being damaged under the conditions above.

## 3. Experimental Results and Analysis

**Figure 3:**
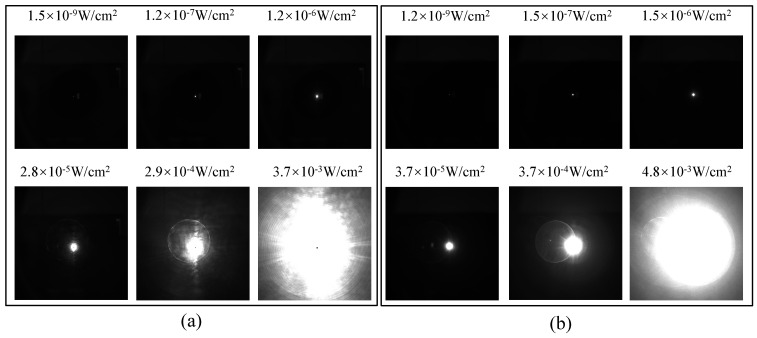
(**a**) Dazzling spots captured by the FSI−CMOS; (**b**) dazzling spots captured by the BSI−CMOS.

The impact of the laser irradiation on the CMOS camera images is illustrated in Figure 3, where Figure 3a is the dazzling spots captured by the FSI-CMOS, and Figure 3b is by the BSI-CMOS CMOS. The exposure time of the camera is set to 6 ms.

We can see from Figure 3 that the laser dazzling images in CMOS cameras were in round-shape, and the size of the image spots went up as the laser power density at the entrance pupil increased. For example, under an irradiation of about $10^{-9}\;{W/\mathrm{cm}}^{2}$, the dazzling spots area is very small, occupying only about 10 to 30 saturated pixels; when it increases to $1\times 10^{-6}\;{W/\mathrm{cm}}^{2}$, the number of saturated pixels approaches around 1000. The saturated spots almost occupy the entire screen at the power density of $1.2\times 10^{-2}\;{W/\mathrm{cm}}^{2}$ for FSI-CMOS and $1.5\times 10^{-2}\;{W/\mathrm{cm}}^{2}$ for BSI-CMOS. The full-screen saturation threshold for the BSI-CMOS was 25% higher than the FSI-CMOS, which indicates that BSI-CMOS has a better capability of laser dazzle protection. Particularly, the FSI-CMOS exhibited over-saturated spots, which might relate to the correlated double sample (CDS) circuit structure of the camera [33], and we can take it as normal saturation during data processing.

**Figure 4:**
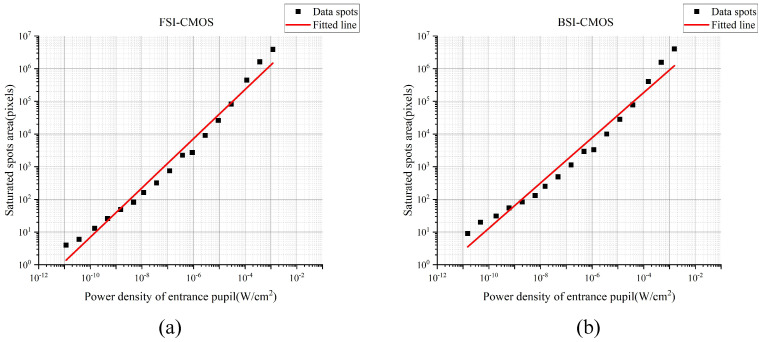
(**a**) The linear fitting was performed for a FSI−CMOS with a slope of 0.73; (**b**) the linear fitting was performed for a BSI−CMOS with a slope of 0.66.

In data processing, we first converted the captured images into gray-scale matrices and then counted the number of saturated pixels. Then, we process the over-saturated pixels into saturated pixels. Subsequently, we plot the diagram of the saturated spots area by pixel number versus the laser irradiation by entrance pupil power density. Finally, we obtained the slope of the lines by performing linear fitting on the scatter plots. Figure 4 shows the fitting results of FSI-CMOS and BSI-CMOS.

Figure 4a,b show the linear fitting results of saturated spots area and power density in double logarithmic coordinates for FSI-CMOS and BSI-CMOS, with the slope at 0.73 ($R^{2}$ = 0.98) and 0.66 ($R^{2}$ = 0.99), respectively. The slope of FSI-CMOS is larger than BSI-CMOS. The reason might be that for FSI-CMOS, the optical signal travels through the metal wires before reaching the photo-diode, and therefore causing stronger scattering. In contrast, the diffraction dominates in BSI-CMOS.

## 4. Simulation Methods and Results

**Figure 5:**
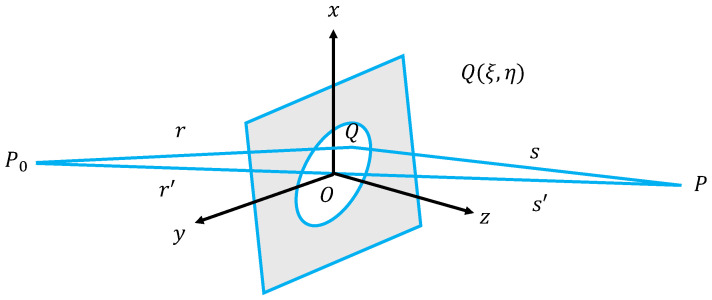
Fresnel–Kirchhoff diffraction diagram.

In order to establish the numerical model of the laser irradiation, we begin with the Fresnel–Kirchhoff diffraction integral expression [34]. Figure 5 presents the diffraction of an aperture on a planar screen, using a Cartesian coordinate system with the origin at the center of the aperture, which is also the diffraction screen. The x-axis and y-axis lie in the plane of the aperture, and the positive z-direction points towards the half-space where the observation point *P* is located. $P_{0}$ is a point source, *Q* is any point on the diffracting circular aperture, and point *P* is the observation point where $P_{0}$’s light passes through point *Q* on the circular aperture.

At position *P*, the Fresnel–Kirchhoff integral expression can be written as [34]

(2) $$U\left(P\right)=-\frac{i\cos\delta }{\lambda }\frac{Ae^{ik(r^{\prime }+s^{\prime })}}{r^{\prime }s^{\prime }}\;\int \int \;e^{ikf(\xi ,\eta )}d\xi d\eta$$

where U(P) represents the complex amplitude at *P* after diffraction. δ is the angle between the line $P_{0}P$ and the normal of the diffraction screen, *A* is a constant, $r^{\prime }$ and $s^{\prime }$ are the distances of $P_{0}$ and *P* to the origin, respectively, and the coordinate of point *Q* is expressed as Q(ξ,η). The expression for f(ξ,η) is given by [34]

(3) $$f\left(\xi ,\eta \right)=-\frac{x_{0}\xi +y_{0}\eta }{r^{\prime }}-\frac{x\xi +y\eta }{s^{\prime }}+\frac{\xi^{2}+\eta^{2}}{2r^{\prime }}+\frac{\xi^{2}+\eta^{2}}{2s^{\prime }}-\frac{{\left(x_{0}\xi +y_{0}\eta \right)}^{2}}{2r^{\prime 3}}-\frac{{\left(x\xi +y\eta \right)}^{2}}{2s^{\prime 3}}\cdots$$

Let $\left(l_{0},m_{0}\right)$ and (l,m) represent the first two directional cosines of $r^{\prime }$ and $s^{\prime }$:

(4) $$\begin{array}{cc} l_{0}=-\frac{x_{0}}{r^{\prime }}, & \;l=\frac{x}{s^{\prime }}\\ m_{0}=-\frac{y_{0}}{r^{\prime }}, & \;m=\frac{y}{s^{\prime }} \end{array}$$

Then, Equation (3) can be written in the following form:

(5) $$f\left(\xi ,\eta \right)=\left(l_{0}-l\right)\xi +\left(m_{0}-m\right)\eta +\frac{1}{2}\left[\left(\frac{1}{r^{\prime }}+\frac{1}{s^{\prime }}\right)\left(\xi^{2}+\eta^{2}\right)-\frac{{\left(l_{0}\xi +m_{0}\eta \right)}^{2}}{r^{\prime }}-\frac{{\left(x\xi +y\eta \right)}^{2}}{s^{\prime }}\right]\ldots$$

When the quadratic and higher terms of ξ and η in function *f* can be neglected, the situation is called Fraunhofer diffraction. Normally, it happens when both the light source and the observation point are at positive infinity. In this situation, the following expressions should be satisfied:

(6) $$|r^{\prime }|\gg \frac{{\left(\xi^{2}+\eta^{2}\right)}_{\max}}{\lambda }\;\mathrm{and}\;\left|s^{\prime }\right|\gg \frac{{\left(\xi^{2}+\eta^{2}\right)}_{\max}}{\lambda }$$

Otherwise, the Fraunhofer diffraction formula cannot be used and the quadratic term has to be taken into account, which is called Fresnel diffraction. In our experiments, the laser may not be strictly parallel where Equation (6) cannot be satisfied, so it is necessary to take the quadratic term into account. Therefore, the expression of U(P) can be re-written as

(7) $$U\left(P\right)=C\;\int \int \;e^{ik\left\{\left(l_{0}-l\right)\xi +\left(m_{0}-m\right)\eta +\frac{1}{2}\left[\left(\frac{1}{r^{\prime }}+\frac{1}{s^{\prime }}\right)\left(\xi^{2}+\eta^{2}\right)-\frac{{\left(l_{0}\xi +m_{0}\eta \right)}^{2}}{r^{\prime }}-\frac{{\left(x\xi +y\eta \right)}^{2}}{s^{\prime }}\right]\right\}}d\xi d\eta$$

where *C* represents the constant in front of the integral, with the expression as follows:

(8) $$C=\frac{1}{\lambda R}\sqrt{\frac{P_{L}}{D}}$$

In Equation (8), λ represents the wavelength of light, *R* denotes the distance from point *O* to the intersection of line $P_{0}O$ with the observation plane, $P_{L}$ is the power of the light after collimation, and *D* represents the area of the diffraction aperture.

It is difficult to derive the analytical solution for Equation (7), so we need to use numerical integration to calculate *U(P)*. Then, the laser density *I(P)* can be calculated according to Equation (9).

(9) $$I\left(P\right)={\left|U\left(P\right)\right|}^{2}$$

**Figure 6:**
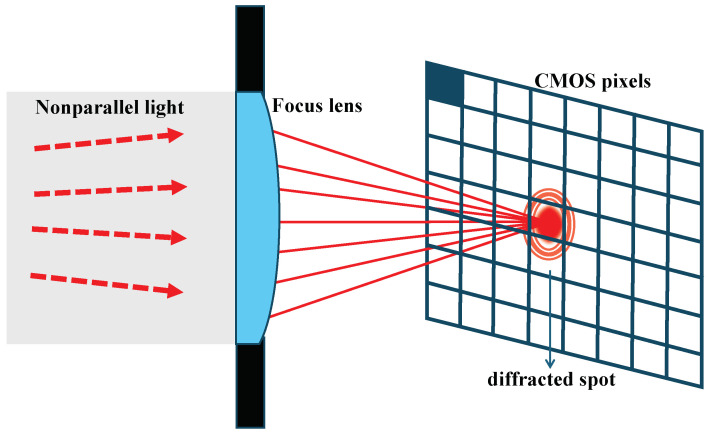
Schematic of Fresnel diffraction with the addition of a focusing lens.

The numerical model was established mainly based on Equations (7) and (8), and the corresponding physical process can be described as shown in Figure 6. If a well-corrected lens is placed behind the diffraction screen, non-parallel light passing through the lens can be converged into the image plane, representing the Fresnel diffraction phenomenon (instead of Fraunhofer diffraction for parallel light). As the lens is introduced, *R* in Equation (7) becomes equivalent to the focal length *F*. $r^{\prime }$ is approximately infinite, and therefore $l_{0}$ and $m_{0}$ can be considered as zero according to Equation (4). So, in the numerical integration, we only need to assign values to *l* and *m*. Furthermore, because of the symmetry of the diffraction image, only one direction is needed to be calculated (either *l* or *m*).

In the simulation, we set eight different orders of magnitude for the power density and corresponding parameters, as detailed in Table 3. These parameters are consistent with those in the experimental section.

**Figure 7:**
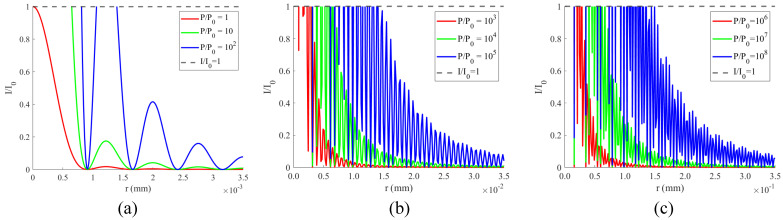
(**a**) Distribution of diffracted intensity on the image plane at power density *P* of $P_{0}$, 10$P_{0}$ and 10^2^$P_{0}$; (**b**) distribution of diffracted intensity on the image plane at power density *P* of 10^3^$P_{0}$, 10^4^$P_{0}$ and 10^5^$P_{0}$; (**c**) distribution of diffracted intensity on the image plane at power density *P* of 10^6^$P_{0}$, 10^7^$P_{0}$ and 10^8^$P_{0}$.

We calculated the diffraction intensity distributions at different power densities, as shown in Figure 7. We take $P_{0}$ as a reference, which represents the laser power density that only one single-pixel is saturated, and the corresponding image intensity $I_{0}$ is set as 1. If the image intensity *I* at a particular location is larger than $I_{0}$, the corresponding pixel saturates. Figure 7a shows the distribution of diffracted intensity on the image plane at power density *P* of $P_{0}$, 10$P_{0}$, and 10^2^$P_{0}$; Figure 7b presents the trend of the diffracted intensity distribution at power density *P* of 10^3^$P_{0}$, 10^4^$P_{0}$, and 10^5^$P_{0}$; and Figure 7c presents the trend of the diffracted intensity distribution at power density *P* of 10^6^$P_{0}$, 10^7^$P_{0}$ and 10^8^$P_{0}$. We can see that as *P* increases, the portion where *I*/ $I_{0}$ > 1 is evidently increasing.

**Figure 8:**
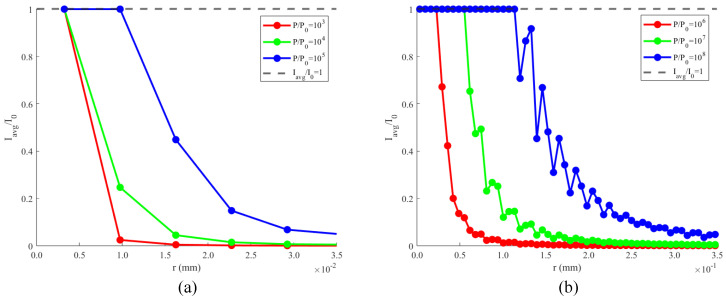
(**a**) Average relative intensity distribution for *P* of 10^3^$P_{0}$, 10^4^$P_{0}$, and 10^5^$P_{0}$; (**b**) average relative intensity distribution for *P* of 10^6^$P_{0}$, 10^7^$P_{0}$, and 10^8^$P_{0}$.

After calculating the diffracted intensity distributions, we need to select the pixel points at which the relative diffracted intensity *I*/$I_{0}$ is greater than 1, taking them as saturated points. Then, we averaged the diffraction intensity in pixel size. The new diffracted intensity distribution map is shown in Figure 8. When $I_{avg}/I_{0}=1$, the radius *r* is considered as the radius of saturated laser spots $r_{sat}$. The area of the dazzling spots *S* can be calculated accordingly.

**Figure 9:**
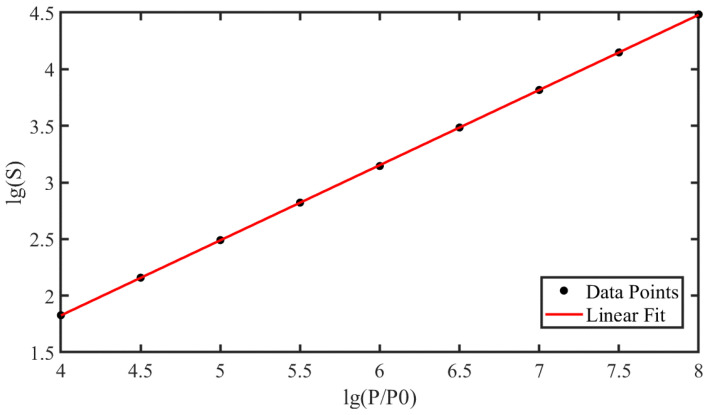
The linear fit of the dazzling spots area in relation to power density; the slope of the fitted line is 0.64.

We plotted the relationship between the area of the dazzling spots *S* and *P*/$P_{0}$ under different power density, as shown in Figure 9. In a double logarithmic coordinate, a linear relationship is observed, where the slope of the fitted line is 0.64. The obtained expression is

(10) $$lg\left(S\right)=0.64\mathrm{lg}\left(\frac{P}{P_{0}}\right)-0.52$$

where the $R^{2}$ of the fitted line is 0.999, indicating a good linear relationship between the two variables.

The simulation results demonstrate that under Fresnel diffraction conditions, there is a linear relationship between the relative laser power density *P*/$P_{0}$ and the area of the saturated spots *S* with the slope of 0.64 in double logarithmic coordinates. This matches the experimental results for the BSI-CMOS (the slope of the fitting is 0.66), keeping the error relative to the theoretical value around 3%. However, the slope of the experimental fitting for the FSI-CMOS is 0.73, and the error relative to the theoretical value is around 14%. Scattering is likely to be involved in FSI-CMOS [25]. We conclude that the scattering is less effected in BSI-CMOS structure, as the light can shine on the photo-diode directly without being blocked by the metal lines or other circuits of the chips.

## 5. Conclusions

In this paper, we investigated the laser dazzling spots of typical FSI-CMOS and BSI-CMOS cameras under the radiation of a 532 nm CW laser, and gave the explanation of the phenomenon based on the Fresnel diffraction model. There is a liner relationship between power density of entrance pupil and saturated spots area in double logarithmic coordinates for CMOS in our experiment, and the slope is 0.73 for FSI-CMOS and 0.66 for BSI-CMOS. The Fresnel diffraction model results show that there is a linear relationship between the relative laser power density $P/P_{0}$ and the area of the saturated spots *S* with the slope of 0.64 in double logarithmic coordinates. The experimental results for the BSI-CMOS matches the simulation results with an error of 3%, while the FSI-CMOS is beyond the simulations with an error margin of 14%. The results show that in FSI-CMOS, the scattering is mainly involved. Overall, the study provides a relevant reference for studying laser dazzle and offers data support for improving CMOS performance under high laser irradiation.

## Figures and Tables

**Table 1:** Relevant parameters of 532 nm CW laser.

Laser Parameters	Value	Unit of Parameters
Wavelength	532 ± 1	nm
Spectral Line Width	<1	nm
Output Power	0–2500	mW
Transverse Mode	TEM_00_	/
Beam Quality (M^2^ factor)	<1.5	/
Polarization	Line Polarization	/
Polarization Ratio	>100:1	/
Beam Diameter at Aperture	3	mm
Beam Divergence (full angle)	<1.5	mrad
Power Supply	90–240 VAC@50 Hz	/
Operation Temperature	10–35	°C

**Table 2:** Partial introduction of parameters for two types of cameras.

Sensor Type	Focal Plane Size	Resolution	Pixel Size	Bit Depth
FSI CMOS	13.3 mm × 13.3 mm	2048 × 2048	6.5 μm × 6.5 μm	16
BSI CMOS	13.3 mm × 13.3 mm	2048 × 2048	6.5 μm × 6.5 μm	16

**Table 3:** Simulation parameters.

Wavelength	Lens Focal Length	Circular Aperture Diameter	Power Range
532 nm	35 mm	25 mm	$10^{-10}\;W\;\mathrm{to}\;10^{-2}\;W$

## Data Availability

The original contributions presented in the study are uploaded to GitHub and made it publicly available.The website for the dataset is: https://github.com/Rains0502/Experimental-database.git (accessed on 2 September 2024).

## Abbreviations

The following abbreviations are used in this manuscript:

CMOS	Complementary Metal Oxide Semiconductor
CCD	Charge Coupled Device
WFOV	Wide Field of View
UAVs	Unmanned Aerial Vehicles
CW	Continuous Wave
FSI-CMOS	Front Side Illumination CMOS
BSI-CMOS	Back Side Illumination CMOS
OD	Optical Density
CDS	Correlated Double Sample
